# Anterior cruciate ligament reconstruction: effect of graft type and gender on early to mid-term clinical outcomes

**DOI:** 10.1007/s12306-024-00824-2

**Published:** 2024-05-29

**Authors:** O. Mann, O. Al-Dadah

**Affiliations:** 1 The Medical School, Newcastle University, Framlington Place, Newcastle-Upon-Tyne, NE2 4HH UK; 2 Department of Trauma and Orthopaedic Surgery, South Tyneside District Hospital, Harton Lane, South Tyneside, NE34 0PL UK; 3 Translational and Clinical Research Institute, Faculty of Medical Sciences, Newcastle University, Framlington Place, Newcastle-Upon-Tyne, NE2 4HH UK

**Keywords:** Anterior cruciate ligament reconstruction, Patient-reported outcome measures, Patella tendon, Hamstring tendon, Autograft, Gender

## Abstract

**Introduction:**

Anterior cruciate ligament (ACL) rupture is a debilitating condition and often requires surgery to restore joint stability. Common autografts used for reconstruction include patella tendon and hamstring tendons. The primary aim of this study was to evaluate the early to mid-term clinical outcomes of ACL reconstruction using validated patient-reported outcome measures (PROMs). The secondary aim was to compare clinical outcomes between patella tendon and hamstring tendon autografts. The tertiary aim was to compare clinical outcomes between males and females.

**Methods:**

Patients with an ACL rupture were evaluated before and after surgery using PROM scores which included Lysholm, Tegner, International Knee Documentation Committee (IKDC), Knee Injury and Osteoarthritis Outcome Score (KOOS), Short Form-12 Item (SF-12) and EQ-5D-5L.

**Results:**

A total of 87 patients were included in the study. All PROM scores significantly improved following surgery (p < 0.001) at a mean follow-up time of 28 months (range 12 to 88 months). The patella tendon subgroup (n = 27) had superior post-operative results as compared to the hamstring tendon subgroup (n = 60) for KOOS sport and recreation (p = 0.005), KOOS quality of life (p = 0.025), KOOS overall (p = 0.026), Tegner (p = 0.046) and IKDC (p = 0.021) scores. There was no significant difference of PROM scores between males (n = 60) and females (n = 27) (p > 0.05).

**Conclusions:**

ACL reconstruction significantly improves clinical outcomes for patients with symptomatic instability consequent to ACL rupture. Overall, patella tendon autograft resulted in better clinical outcomes as compared to hamstring tendon autograft following surgery. Gender did not influence clinical outcome following ACL reconstruction.

## Introduction

Anterior cruciate ligament (ACL) rupture is a debilitating injury which can result in recurrent episodes of knee joint instability and is becoming increasingly prevalent. Consequently, the volume of surgical reconstruction of the ACL in clinical practice is rising too. In England alone, it is estimated that around 15,000 primary ACL reconstructions are performed every year [1]**.** Current national guidelines in the United Kingdom (UK) state that the main indication for ACL reconstruction is symptomatic instability, and that all patients should be offered prehabilitation prior to their procedures [2]. In most cases, surgery is considered following an initial period of conservative treatment (i.e. physiotherapy) in an attempt to strengthen the peri-articular muscles and provide further stability for the ACL deficient knee. The decision between early surgical reconstruction or a protracted trial of conservative treatment is based on patient preference following an informed decision made in discussion with the surgeon. If recurrent instability persists despite an initial trial of conservative treatment, surgery is then considered. ACL reconstruction can return 65% of patients to the same level of sporting performance following their procedure [3]. The two commonest autografts used for arthroscopic surgical reconstruction include the hamstring tendons (HT), and the patella tendon, also known as a bone-patella tendon-bone (BTB) autograft [4]. The knee joint must be able to go through a full range of movement as a prerequisite to either procedure [2, 5].

These two surgical techniques have well-established advantages and disadvantages. The BTB autograft is thought to be stronger as the natural bone-tendon attachment is very strong, with the potential for bone integration [6], and results in less knee laxity than a hamstring tendon autograft [7]. However, patients having BTB autografts sometimes complain of more severe anterior knee pain post-operatively than those receiving HT autografts [6]. Therefore, patients who do a lot of kneeling in their occupation or for cultural, religious or recreational reasons will tend to receive HT autografts as anterior knee pain would be more debilitating for them [8]. The BTB autograft is thought to have a higher rate of complications, including patella fractures, patella tendon rupture and quadriceps weakness [9, 10]. The HT autograft, although thought to be weaker, can be used to avoid these complications, whilst maintaining the extensor mechanism of the lower limb [6]. Some of the disadvantages of the HT autograft includes a longer biological integration time due to the lack of bone plugs, weakness of the hamstring musculature which means there is reduced stabilisation of the knee joint, potentially making re-rupture more likely [6].

The primary aim of this study was to evaluate the early to mid-term clinical outcomes of ACL reconstruction using validated patient-reported outcome measures (PROMs). The secondary aim was to compare clinical outcomes between patella tendon and hamstring tendon autografts. The tertiary aim was to compare clinical outcomes between males and females. The first hypothesis of this study is that surgical reconstruction will improve clinical outcomes in patients with ACL rupture. The second hypothesis is that no difference exists in terms of clinical outcomes between the two autografts used for surgery. The third hypothesis is that no difference exists between genders regarding clinical outcomes following ACL reconstruction.

## Methods

This is a longitudinal observational study. All the patients included in this study attended a specialist knee clinic and underwent ACL reconstruction surgery between 2015 and 2022 following clinical assessment and radiological investigation. This study was exempt from Institutional Review Board (IRB)/Ethics Committee approval as it was a pragmatic study evaluating the existing clinical practice of the senior author (consultant orthopaedic surgeon). This study was registered with the hospital’s Clinical Effectiveness Department (registration number CA10358). This therapeutic research study constituted part of the first author’s Masters dissertation.

Inclusion criteria consisted of patients with an ACL tear whose symptoms where refractory to an initial period of conservative treatment (i.e. physiotherapy, activity modification, etc.) who subsequently underwent an arthroscopic primary, single-bundle ACL reconstruction using either BTB or HT autografts. Exclusion criteria consisted of posterior cruciate ligament (PCL) tear, multi-ligament reconstruction, revision ACL reconstruction and advanced osteoarthritis.

### Surgical technique

All patients underwent arthroscopic ACL reconstruction using the anatomic single-bundle technique and subsequently the same structured post-operative physiotherapy rehabilitation programme. Returning back to contact sports was only permitted 12 months post-operatively.

The HT autograft surgery involves harvesting tendons of the gracilis and semitendinosus muscles. A small incision is made over the hamstring insertion (pes anserinus), and the tendons are stripped from the muscle. The hamstring distal insertions on the pes anserinus were preserved, and the tendons were only detached from their proximal musculotendinous junction using a tendon stripper. Once harvested they are whip stitched and then folded over to create a quadrupled hamstring tendon graft. Tunnels (corresponding in size to the harvested graft) are then drilled into the femur (via the transportal technique) and the tibia (using a tibial jig set to 55 degrees), both of which are centred on the native ACL footprint. The graft is then pulled through the tunnels and secured to the femur via suspensory fixation using EndoButton (Smith & Nephew Inc., Andover, Massachusetts, USA) and to the tibia using either polyetheretherketone (PEEK) interference screws (Smith & Nephew Inc., Andover, Massachusetts, USA) or round cannulated interference (RCI) screws (Smith & Nephew Inc., Andover, Massachusetts, USA). The graft was tensioned with the knee in full extension.

The BTB autograft surgery involves an anterior vertical incision centred over the patella tendon. The middle third (10 mm width) of the entire length of the tendon is harvested (sharp dissection with scalpel) along with its attached proximal and distal bone wedges (10 mm width × 20 mm length) from the patella and the tibial tuberosity, respectively, using a miniature oscillating saw. Tunnels (corresponding in size to the bone wedges) are then drilled into the femur (via the transportal technique) and the tibia (using a tibial jig set to 60 degrees), both of which are centred on the native ACL footprint. The graft is then pulled through the tunnels and secured using Softsilk interference screws (Smith & Nephew Inc., Andover, Massachusetts, USA) both in the femur and the tibia. The graft was tensioned with the knee in full extension.

### Clinical outcome scores

A total of 6 validated patient-reported outcome measures (PROMs) were used in this study which included the Knee Injury and Osteoarthritis Outcome Score (KOOS) [11, 12], EuroQol-5 Dimension-5 level (EQ-5D-5L) [13–16], International Knee Documentation Committee (IKDC) score [17, 18], Tegner score [19], Lysholm score [19] and the 12-item Short Form survey (SF-12) [20]. PROM data was collected pre-operatively (at the time of the patients' initial outpatient clinic appointment) and post-operatively (the latest point of contact at the time of conducting this study via postal questionnaire). Some of the PROMs have been validated and used in children, including the paeds-IKDC [21, 22] and paediatric KOOS [23, 24]. The Tegner score can also be used in children as it a measure of activity level. Of the 87 patients included in the study, there were two patients under 16 who completed the paediatric PROMs pre-operatively, and then the adult PROMs post-operatively (when aged over 16). There was one patient with missing pre-operative data, who completed the paediatric PROMs post-operatively. There were two patients who completed the pre-op paediatric PROMs but were lost to follow-up post-intervention, and so have no post-op data. These paediatric scores were included in overall data analysis.

### Statistical analysis

Plotted histograms with fitted curve lines, box-plots, normal Q-Q plots, and the Shapiro–Wilk statistic were used to test normality of data distribution. All the PROM data (continuous variables) displayed a skewed distribution and therefore the relevant nonparametric statistical tests were used for the data analysis. The level of statistical significance was set at *p* < 0.05. Statistical analysis was performed using SPSS for Windows version 28.0 (IBM Corp., Armonk, New York).

## Results

**Fig. 1:**
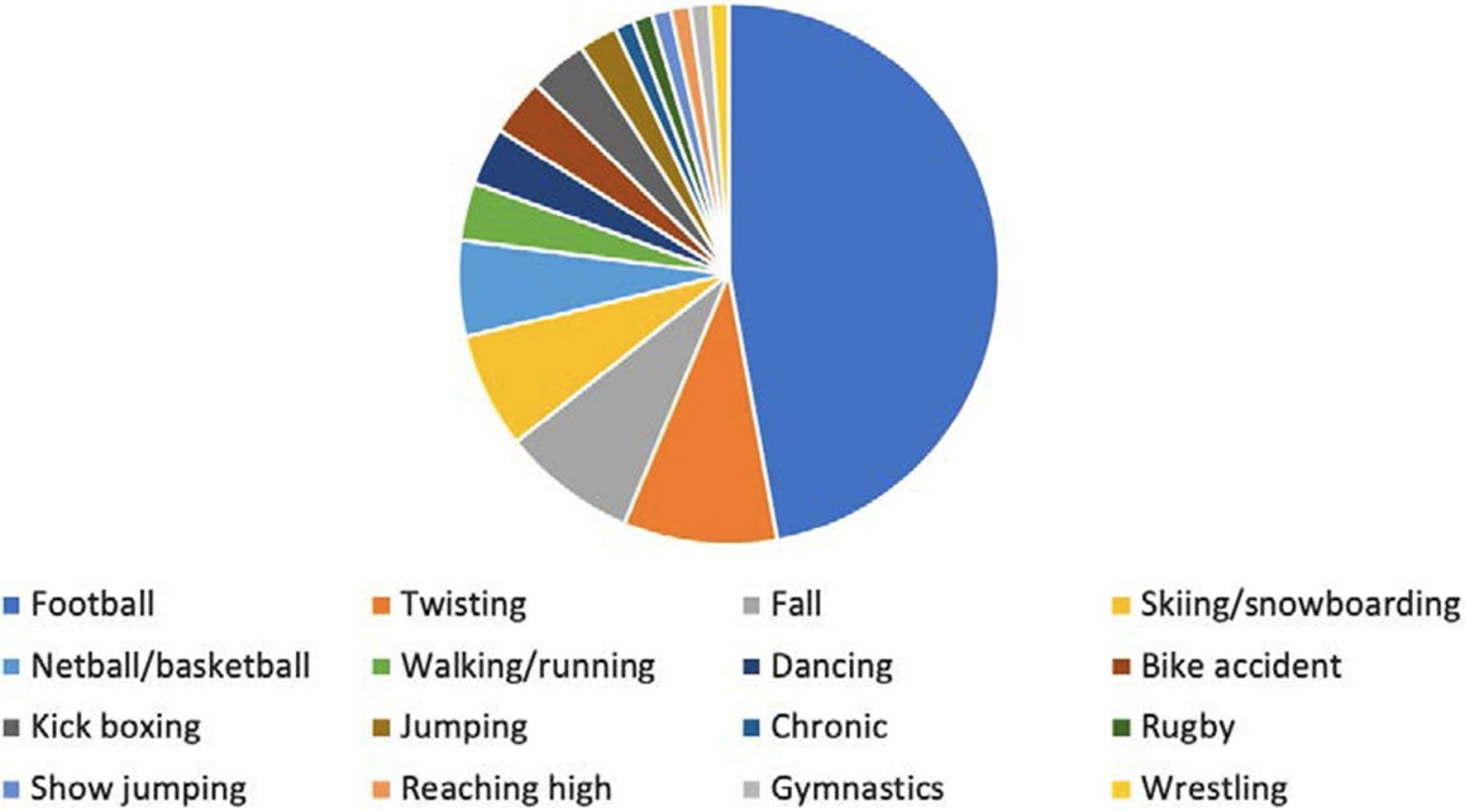
Mechanism of injury of all patients (*n* = 87)

Table 1 shows the demographics of all the patients included in the study, showing a mean age of 31.5 years, with over twice as many males as females (60:27), and a mean BMI of 27.5. The disproportionately higher number of males reflects the participation in higher risk activities which commonly generate ACL injuries. Figure 1 shows the mechanism of injury of all participants in the study, with the highest number of patients injuring their ACL playing football (*n* = 41), followed by twisting (*n* = 8), falling (*n* = 7), and skiing/snowboarding (*n* = 6). The mean time between injury and surgery was 24 months.

**Table 1:** Patient demographics of entire study cohort

	(*n* = 87)
Age (years) (mean (range))	31.5 (12–62)
Sex (male: female)	60:27
Laterality (left: right)	44:43
Height (cm) (mean (SD))	174.3 (8.8)
Weight (kg) (mean (SD))	84.1 (18.0)
BMI (kg/m^2^) (mean (SD))	27.5 (5.4)
Associated meniscus tear (yes: no)	70:17
Meniscus tear location (medial: lateral)	51:38

SD, standard deviation; BMI, body mass index

### Clinical outcome scores

Post-operative PROM score completion timeframe was a mean of 28 months (range 12–88). Of the 87 cases that were contactable post-operatively, there were 2 ACL grafts that had re-ruptured (2.4%), one of which was a hamstring tendon graft, and the other a BTB graft. Table 2 shows that all the PROM scores (except the SF-12 MCS subscore) showed a significant longitudinal improvement (*p* < 0.001) from pre-operative to post-operative results. The Tegner scores represented in this table are Tegner post-injury to Tegner post-operatively. Table 3 shows that the Tegner score changed significantly between all three points of measurement. Pre-injury to post-injury showed a significant decrease (p < 0.001) in activity level associated with ACL rupture, pre-injury to post-op still showed a significant decrease (*p* < 0.001) implying surgery did not restore patients to their pre-injury sporting activity levels. Post-injury to post-op (*p* < 0.001) showed a significant increase, indicating that ACL reconstruction is beneficial to restore knee function which allowed for an improvement in activity level.

**Table 2:** Comparison of pre-operative PROMs versus post-operative PROMs of entire study cohort (*n* = 87)

	Pre-op median (IQR)	Post-op median (IQR)	*p* value^a^	*Z*
KOOS pain	61 (50–74)	89 (78–97)	< 0.001*	−5.7
KOOS symptoms	57 (43–68)	79 (68–86)	< 0.001*	−4.7
KOOS ADL	71 (56–82)	97 (90–100)	< 0.001*	−5.5
KOOS sport/rec	30 (15–50)	75 (60–85)	< 0.001*	−5.2
KOOS QoL	19 (6–38)	63 (44–75)	< 0.001*	−6.1
KOOS overall	48 (36–61)	81 (70–87)	< 0.001*	−5.8
IKDC	42 (30–53)	75 (64–86)	< 0.001*	−6.2
Lysholm	53 (40–67)	87 (76–91)	< 0.001*	−6.0
Tegner	2 (1–3)	5 (4–7)	< 0.001*	−5.3
EQ-5D index	0.621 (0.445–0.723)	0.837 (0.728–1)	< 0.001*	−5.1
EQ-5D VAS	70 (50–85)	85 (70–90)	< 0.001*	−3.7
SF-12 MCS	51 (41–57)	53 (45–58)	0.367	−0.9
SF-12 PCS	35 (30–41)	54 (47–57)	< 0.001*	−5.5

IQR, inter-quartile range, PROM, patient-reported outcome measures; BTB, bone-patella tendon-bone; HT, hamstring tendon; KOOS, knee injury and osteoarthritis outcome score; ADL, activities of daily living; Sport/Rec, sport and recreation function; QoL, quality of life; IKDC, international knee documentation committee score; SF-12, short form 12; MCS, mental component summary; PCS, physical component summary; EQ-5D, EuroQol-5D; VAS, visual analogue score

^a^Wilcoxon signed rank test

^*^Statistically significant < 0.05

**Table 3:** Comparison of Tegner activity scores of entire study cohort (*n* = 87)

Pre-injury median (IQR)	Post-injury median (IQR)	Post-op median (IQR)	*p* value^a^	*Z*
8 (7–9)	2 (1–3)		< 0.001*	−7.1
8 (7–9)		5 (4–7)	< 0.001*	−5.8
	2 (1–3)	5 (4–7)	< 0.001*	−5.3

IQR, inter-quartile range

^a^Wilcoxon signed rank test

^*^Statistically significant < 0.05

### Graft comparison

Table 4 shows the demographics of patients in the BTB group and the HT group. The BTB group were slightly older than the HT group. All the BTB group were male patients, whereas the HT group had a more balanced distribution of males and females. The senior author favoured a hamstring autograft for female patients as it resulted in a smaller skin scar and therefore more aesthetically acceptable whilst also restoring knee joint stability.

**Table 4:** Patient demographics of BTB group versus HT group

	BTB group (*n* = 27)	HT group (*n* = 60)
Age (years) (mean (range))	34.6 (18–62)	28.8 (12–56)
Sex (male: female)	27:0	33:27
Laterality (left: right)	12:15	32:28
Height (cm) (mean(SD))	179.9 (7.3)	171.9 (8.1)
Weight (kg) (mean(SD))	93.4 (16.3)	80.4 (17.6)
BMI (kg/m^2^) (mean(SD))	28.7 (4.3)	27.1 (5.9)
Associated meniscus tear (yes: no)	22:5	48:12
Meniscus tear location (medial: lateral)	15:13	36:25

SD, standard deviation; BMI, body mass index; BTB, bone-patella tendon-bone; HT, hamstring tendon

Table 5 shows that overall, there was no significant difference between the BTB group and the HT group pre-operatively (except for the EQ-5D index score only, *p* = 0.044). Table 5 also shows that the BTB group scored higher than the HT group for KOOS sport and recreation (*p* = 0.005), KOOS quality of life (*p* = 0.025), KOOS overall (*p* = 0.026), Tegner score (*p* = 0.046) and the IKDC score (*p* = 0.021). Overall, the BTB group demonstrated superior results as compared to the HT group.

**Table 5:** Between group comparison of pre-operative and post-operative PROMs: BTB group versus HT group

	Pre-operative					Post-operative				
	BTB group (*n* = *27*) median (IQR)	HT group (*n* = *60*) median (IQR)	*p* value^a^	*Z*	*U*	BTB group (*n* = *27*) median (IQR)	HT group (*n* = *60*) median (IQR)	*p* value^a^	*Z*	*U*
KOOS pain	61(53–75)	58(46–74)	0.345	−0.9	455	92(77–100)	86(77–94)	0.331	−1.0	279
KOOS symptoms	64(51–74)	54(41–68)	0.112	−1.6	453	82(71–86)	79(67–87)	0.666	−0.4	318
KOOS ADL	72(61–81)	71(48–85)	0.585	−0.5	542	98(90–100)	94(86–100)	0.284	−1.1	274
KOOS sport/rec	35(19–50)	30(18–50	0.804	−0.2	477	85(75–95)	75(48–80)	0.005*	−2.8	167
KOOS QoL	19(6–38)	19(6–38)	0.577	−0.6	486	72(56–88)	56(35–69)	0.025*	−2.2	209
KOOS overall	50(43–60)	48(35–61)	0.530	−0.6	448	84(76–94)	80(64–83)	0.026*	−2.2	195
IKDC	38(29–47)	43(30–56)	0.488	−0.7	475	82(71–89)	72(57–77)	0.021*	−2.3	204
Lysholm	57(42–67)	49(40–67)	0.601	−0.5	476	89(81–93)	86(72–90)	0.144	−1.5	230
Tegner	2(2–3)	2(1–3)	0.405	−0.8	339	6(5–7)	5(3–6)	0.046*	−2.0	191
EQ-5D index	0.708 (0.428–0.781)	0.604 (0.471–0.670)	0.044*	−2.0	371	0.837 (0.724–1)	0.795 (0.700–1)	0.566	−0.6	277
EQ-5D VAS	70(50–88)	65(50–75)	0.447	−0.8	459	90(75–90)	80(65–90)	0.232	−1.2	251
SF-12 MCS	51(40–58)	48(40–55)	0.390	−0.9	418	53(44–58)	53(46–59)	0.897	−0.1	283
SF-12 PCS	38(31–46)	34(29–39)	0.145	−1.5	375	55(50–57)	52(42–56)	0.136	−1.5	215

IQR, inter-quartile range; PROM, patient-reported outcome measures; BTB, bone-patella tendon-bone; HT, hamstring tendon; KOOS, knee injury and osteoarthritis outcome score; ADL, activities of daily living; Sport/Rec, sport and recreation function; QoL, quality of life; IKDC, International knee documentation committee score; SF-12, short form 12; MCS, mental component summary; PCS, physical component summary; EQ-5D, EuroQol-5D; VAS, visual analogue score

^a^Mann-Whitney U test

^*^Statistically significant < 0.05

### Gender comparison

Table 6 shows that overall, there were no significant differences between males and females pre-operatively. Although there was a statistically significant difference for the Tegner score between the two genders (*p* = 0.012), this did not amount to a clinically significant difference as they both scored median 2.

**Table 6:** Between group comparison of pre-operative and post-operative PROMs: males versus females

	Pre-operative					Post-operative				
	Male group (*n* = *60*) median (IQR)	Female group (*n* = *27*) median (IQR)	*p* value^a^	*Z*	*U*	Male group (*n* = *60*) median (IQR)	Female group (*n* = *27*) median (IQR)	*p* value^a^	*Z*	*U*
KOOS pain	61 (53–75)	57 (41–64)	0.156	−1.4	480	89 (77–98)	91 (83–97)	0.592	−0.5	370
KOOS symptoms	61 (50–71)	43 (36–68)	0.096	−1.7	509	79 (68–86)	82 (68–89)	0.443	−0.8	376
KOOS ADL	72 (57–84)	65 (38–76)	0.084	−1.7	502	97 (88–100)	96 (90–99)	0.468	−0.7	358
KOOS sport/rec	35 (20–50)	28 (0–40)	0.150	−1.4	414	75 (65–85)	75 (56–81)	0.292	−1.1	329
KOOS QoL	21 (13–31)	13 (5–38)	0.427	−0.8	535	63 (41–78)	66 (43–69)	0.742	−0.3	384
KOOS overall	50 (41–61)	42 (27–54)	0.077	−1.8	387	81 (70–88)	82 (73–84)	0.786	−0.3	379
IKDC	45 (32–54)	36 (22–45)	0.055	−1.9	435	76 (66–87)	72 (64–85)	0.442	−0.8	355
Lysholm	57 (43–68)	46 (31–64)	0.091	−1.7	438	86 (74–93)	88 (78–90)	0.962	−0.0	384
Tegner	2 (2–3)	2 (1–2)	0.012*	−2.5	276	5 (4–7)	4 (3–6)	0.034*	−2.1	220
EQ-5D index	0.620 (0.454–0.735)	0.623 (0.332–0.661)	0.400	−0.8	521	0.837 (0.733–1)	0.837 (0.679–1)	0.464	−0.7	353
EQ-5D VAS	70 (57–85)	58 (40–76)	0.083	−1.7	444	85 (70–90)	88 (70–91)	0.499	−0.7	353
SF-12 MCS	52 (41–57)	47 (33–57)	0.372	−0.9	449	53 (47–58)	54 (43–59)	0.966	−0.0	358
SF-12 PCS	36 (31–44)	33 (26–39)	0.227	−1.2	424	55 (50–57)	52 (45–57)	0.193	−1.3	283

IQR, inter-quartile range; PROMs, patient-reported outcome measures; BTB, bone-patella tendon-bone; HT, hamstring tendon; KOOS, knee injury and osteoarthritis outcome score; ADL, activities of daily living; Sport/Rec, sport and recreation function; QoL, quality of life; IKDC, international knee documentation committee score; SF-12, short form 12; MCS, mental component summary; PCS, physical component summary; EQ-5D, EuroQol-5D; VAS, visual analogue score

^a^Mann-Whitney U test

^*^Statistically significant < 0.05

Table 6 also shows that only the Tegner score showed a significant difference between the two genders following ACL reconstruction (*p* = 0.034), with males (median 5) scoring higher than females (median 4). None of the other PROM scores showed significant differences between the two genders.

## Discussion

This study demonstrated that ACL reconstruction significantly improves clinical outcomes in patients with symptomatic ACL rupture. Overall, BTB autograft showed significantly better clinical outcomes as compared to HT autografts post-operatively. Gender did not influence clinical outcome following ACL reconstruction.

There was a significant longitudinal improvement from pre-op PROMs to post-op PROMs, indicating that surgical reconstruction is a beneficial procedure for patients with a ruptured ACL. The only exception was the SF-12 MCS which did not show any significant difference as it is mainly a reflection of the patient’s mental health rather than physical function of the knee. PROMs are a reliable method of quantitatively evaluating the subjective function of patients’ knee symptomatology. This has been shown to be the case in previous studies using KOOS, Lysholm and IKDC scores [25, 26]. Other studies have used PROMs to assess factors contributing to favourable outcomes following ACL reconstruction. Randsborg et al. [25] assessed factors associated with poor outcome following ACL reconstruction, but only used IKDC as their sole outcome measure. Cristiani et al. [26] used KOOS and Lysholm scores as their outcome measures in a similar study. The strength of the present study was the use of a wide range of different validated PROMs (both disease specific and generic health scoring systems) to analyse the data collected. Furthermore, the present study also had a longer follow-up interval than many other studies, which is potentially more relevant when counselling patients on the pros and cons of different graft types, and their outcomes.

The post-operative PROM score analysis in the present study showed that patients in the BTB group had greater self-reported knee function when compared to the hamstring tendon group. In contrast, Cristiani et al. [26] found that the KOOS score was significantly better in four out of the five subscores in favour of hamstring tendon recipients as compared to patella tendon grafts, although they too found no significant difference in Lysholm score between the two graft types [26]. Similarly, Hamrin et al. [27] found that improved post-operative KOOS sport and recreation and Tegner scores were associated with hamstring tendon autografts. Randsborg et al. [25] utilised only the IKDC score and found no difference between graft types; however, in the present study the IKDC was significantly higher in the BTB group. The literature is varied as other studies did not find significant differences between graft types which utilised Lysholm, Tegner, IKDC or KOOS [8, 28]. A meta-analysis comprising data pooled from five studies also concluded that there was no significant difference in functional outcomes or knee stability between BTB and HT grafts [29].

Conversely, a review of 16 different meta-analyses found that patients that received a patella tendon graft had superior static knee stability post-operatively, but a higher rate of complications including anterior knee pain and kneeling pain [30]. BTB grafts have shown more favourable return to sport rates [31, 32], lower incidence of graft failure [33] and superior rotational stability [32] in a number of other meta-analyses, but many did find that BTB grafts were associated with a greater risk of complications [30, 32–34]. However, other meta-analyses have found no significant difference in re-rupture rates [8, 31, 32], and one states there is insufficient evidence to draw conclusions relating to functional outcomes [8].

A meta-analysis comparing 15 studies that evaluated the mid-term outcomes of ACL reconstruction, with a minimum 5-year follow-up, found no significant differences in Lysholm, Tegner, IKDC scores, or return to pre-injury activity levels [33]. The present study, as well as previous studies, show variation in outcomes when comparing BTB and hamstring graft types, implying that there may be other factors influencing these outcomes than solely graft choice.

The tensile strength of different grafts has been evaluated [35], showing that the native ACL of a male has a tensile strength of 70.83N, a patella tendon autograft 405.18N and a hamstring tendon autograft 807.07N. From these values, it would be expected that the hamstring tendon autograft would give the most favourable outcomes. A similar study by Noyes et al. [36] showed that the tensile strength of the native ACL is 1725N, and the central portion of the patella tendon has a tensile strength (2900N) that is significantly greater than both the semitendinosus (1216N) and gracilis (838N) tendons. These findings are contrary to those reported by Mert et al. [35] but support the findings of the present study—a BTB graft is associated with improved clinical outcomes when compared to a hamstring tendon graft. This could be due to an increase in tensile strength of the graft providing a greater level of stability to the knee, as well as the bone plug biological integration of the BTB graft within the graft tunnels of the femur and tibia.

Other grafts that have been used include quadriceps tendon (QT) autografts, semitendinosus tendon (without gracilis tendon) autografts, allografts and synthetic grafts; however, the latter is currently not recommended for routine primary ACL reconstruction [2]. QT grafts have been shown to be comparable in functional and clinical outcomes, with similar rates of graft failure in a meta-analysis of 20 observational studies [37]. A meta-analysis of 15 studies comparing the combination of semitendinosus and gracilis tendon hamstring grafts to semitendinosus alone found the PROM scores and knee laxity measurements showed no significant differences between the two techniques [38].

Allografts are sometimes used in revision ACL reconstruction surgeries, more often than in primary reconstruction, particularly if autograft tissue is found to be inadequate [39]. Nissen et al. [40] conducted a study comparing autograft to allograft use for revision ACLR. They found that allograft use was associated with a 2.2 times higher rate of re-revision than autograft use. At 1-year post-op, allograft patients also had greater knee laxity than those who had received an autograft. However, knee function and clinical outcomes were not significantly different between the two groups [40]. Another study also concluded that allografts had a much higher rate of re-rupture, lower sports function, and inferior PROMs than autograft use [41].

When evaluating gender, it is widely accepted that women are more susceptible to ACL ruptures than men [42]. This is speculated to be due to a number of factors, including a greater Q angle, smaller intercondylar notch, neuromuscular performance characteristics, as well as the influence of the menstrual cycle [43]. It has been concluded that static Q angle is not a good predictor of susceptibility to ACL injury [44]. A more accurate predictor of ACL rupture risk is the frontal plane projection angle, formed by two lines connecting the anterior superior iliac spine (ASIS) to the centre of the patella, and another from the centre of the patella to the midpoint between the two malleoli of the ankle joint [44]. Women are known to have a narrower intercondylar notch than men [45], and this in turn has been shown to significantly increase the risk of ACL rupture [46].

Humans have a protective reflex arc in order to the prevent the ACL rupturing, which involves recruitment of the hamstring muscles to prevent anterior translation of the tibia, in response to the ACL being under stress [43]. In a study by Wojtys et al. [47], it was found that females take significantly longer to generate maximum hamstring torque than males, as well as being weaker when this does occur, making women more likely to suffer an ACL rupture. They also found that muscle recruitment in women was also different to that of men, whereby women relied on quadriceps muscle activation for initial knee stabilisation, instead of the hamstring muscles [47].

Hormone fluctuations and the menstrual cycle have been found to put women at risk of ACL rupture. Slauterbeck et al. [48] found that increased serum oestrogen levels resulted in reduced tensile strength of the ACL, making it more likely to tear. Another study also found that during the ovulatory stage of the menstrual cycle, a greater than expected number of ACL injuries occurred, with fewer than expected occurring during the follicular phase of the cycle [49].

Despite the significant variation in susceptibility of ACL rupture, overall a higher number of men present with ACL injuries than women (as seen in the present study) which is further supported in the National Ligament Registry 2022 report, showing that 69% of ACL reconstructions were performed on male patients [50]. This is thought to be due to the fact that men are more likely to take part in high-risk sports or activities such as football or rugby.

Although all the patients in the BTB group were male, the present study found no significant differences between males and females in terms of PROM scores pre-operatively or post-operatively with the only exception being the Tegner score where males had a slightly higher actively level following surgery as compared to females. There are also a number of previous studies evaluating the impact of gender on PROMs. These have shown no significant differences between males and females receiving patella tendon autografts as evaluated by the Tegner score and Lysholm score [42, 51, 52]. Salmon et al. [53] found the same in patients receiving hamstring tendon autografts that there were no significant differences on self-reported knee function. Therefore, it can be concluded that the significant differences observed in the post-operative data analysis between graft types was not influenced by gender. Previous studies have shown that males have better post-operative PROMs than females in IKDC, EQ-5D and KOOS scores [25, 27, 54, 55] which is contrary to the findings of the present study.

The main limitation of this study was the subjectivity of the PROM questionnaires being completed by the patients themselves. Many of the post-operative PROM data was gathered via posting the questionnaires to the patients. The timeframe of returning the completed forms was protracted in some and none at all in others, the latter thereby reduced the total number of patients eligible to be included in this study. Future studies may have a more timely and higher response rate through the use of electronic versions of the PROM questionnaires. A general limitation of all patient-reported outcome measures is the subjective interpretation of the individual items (questions) by the patients of each instrument (questionnaire). All the PROM forms included a brief paragraph instructing patients on how to correctly complete the questionnaires. Despite this, some patients still fill in the forms incorrectly or leave certain parts blank. In some cases, this may mean that overall end scores cannot be produced and hence leads to missing data. This limitation could also be addressed by using an online version of the forms which would only allow a patient to submit the PROM when all items have been correctly and fully completed.

## Conclusion

A significant improvement of clinical outcomes was demonstrated following reconstructive surgery in patients with symptomatic ACL rupture at early to mid-term follow-up. Overall, the outcome of patients who received patella tendon autograft was superior to that of hamstring tendon autograft. Gender did not influence clinical outcome following ACL reconstruction.
